# Ocular Injury by Transient Formaldehyde Exposure in a Rabbit Eye Model

**DOI:** 10.1371/journal.pone.0066649

**Published:** 2013-06-20

**Authors:** Li-Ju Lai, Wei-Hsiu Hsu, Albert M. Wu, June H. Wu

**Affiliations:** 1 Department of Ophthalmology, Chang Gang Memorial Hospital, Chia Yi, Taiwan; 2 Graduate Institute of Clinical Medical Sciences, College of Medicine, Chang Gang University, Kwei San, Tao Yuan, Taiwan; 3 Department of Biochemistry and Molecular Biology, College of Medicine, Chang Gang University, Kwei San, Tao Yuan, Taiwan; 4 Department of Microbiology and Immunology, College of Medicine, Chang Gang University, Kwei San, Tao Yuan, Taiwan; Massachusetts Eye & Ear Infirmary, Harvard Medical School, United States of America

## Abstract

Formaldehyde (FA) is frequently used in sterilizing surgical instruments and materials. Exposure to FA is highly concerned for eye tissues. Rabbit corneal epithelial cells were examined for changes after FA exposure. Our results showed that cell survival decreased 7 days after transient 3 min exposure to more than 100 ppm FA by trypan blue staining while MTT assay detected significant decrease at 20 ppm at 24 hours observation. The decrease of cell survival rate was concentration (up to 600 ppm)- and observation time (1–7 day)- dependent. The cell number decreased after 100 ppm FA exposure for more than 10 min at 7-day observation. The FA treated cells showed increased apoptosis/necrosis and cell cycle accumulation at sub G1 phase as well as mitochondria clustering around nucleus. The *in vivo* rabbit eye exposure for tear production by Schirmer’s test revealed that the FA-induced overproduction of tear also exhibited observation time (1–10 day)- and FA concentration (20–300 ppm for 5 min exposure)-dependent. Activated extracellular signal-regulated kinase (pERK2) in cornea explants by western blotting was reduced and increased c-Jun amino - terminal kinase (JNK) activation (pJNK) in cornea and conjunctiva was evident at 2 month after exposure to 50–200 ppm FA for 5 min. In conclusion, injury to the eye with transient exposure of up to 100 ppm FA for 3 min decreased corneal cell survival while a more sensitive MTT test detected the cell decrease at 20 ppm FA exposure. Morphology changes can be observed even at 5 ppm FA exposure for 3 min at 7 days after. The FA exposure also increased apoptotic/necrotic cells and sub-G1 phase in cell cycle. Long term effect (2 months after exposure) on the eye tissues even after the removal of FA can be observed with persistent JNK activation in cornea and conjunctiva.

## Introduction

Formaldehyde (FA) is a colorless, strong-smelling gas. Commonly used as a preservative in medical laboratories and mortuaries, FA is also found in other products such as chemicals, particle board, household products, glues, permanent press fabrics, paper product coatings, fiberboard, and plywood. It is also widely used as an industrial fungicide, germicide, and disinfectant [1], [2]. In view of its universal use, its volatility and toxicity, contact to FA is a significant concern for human health, especially in the delicate eye tissues. Most common FA exposure was detected among workers in pathology and anatomy laboratories through respiratory tract [3. 4]. Among carcinogen exposure through inhalation pathway, FA was listed as an important chemical [5]. FA was reported to cause adverse reproductive effect, such as spontaneous abortion and leukemia [6], [7]. The toxicity involved chromosome damage, oxidative stress, modified proteins and cellular apoptosis [8], [9]. Interestingly, methanol toxicity was reported through its metabolic intermediate FA [10].

The cornea is the transparent fore portion of the eye that protects the iris, pupil, and anterior chamber and has no blood supply. It has unmyelinated nerve endings that are sensitive to chemicals, temperature and touch. A touch of the cornea results in an automatic reflex to close the eyelid. Oxygen first dissolves in the tears and then diffuses all over the cornea to keep it healthy. Corneal epithelium is a thin epithelial multicellular transparent layer of cells; their moist is preserved with tears. Irregularity or edema of the corneal epithelium induced by many stimulants disrupts the smoothness of the air-tear interface, the most significant constituent of the total refractive power of the eye, thereby reducing visual acuity.

A 37% (12.3 M) FA solution directly contacting the eye can cause severe damage to the cornea and possible blindness [11], [12]. FA released from some materials such as surgical spear (PVA material) used in ophthalmology treatment, is an irritant to the cornea and the ocular surface. The relationship between the FA concentration and the exposure time to the eye with the long-term outcome of ocular damage has not been investigated. In this experiment, we used FA as a chemical to cause injury on rabbit eye or treat primary eye tissue culture and studied the toxicity of FA to the cornea.

## Materials and Methods

### Materials

Dulbecco’s minimum essential medium (DMEM), penicillin-streptomycin, balance salt solution (BSS) and fetal bovine serum (FBS) were from Gibco Life Technologies (Rockville, MD). Trypan blue dye was from Sigma (St. Louis, MO).

### Animal Ethics

Thirty-Six healthy New Zealand White Rabbits (2.0–2.0 Kg) were used in this study. Fifteen rabbits were employed for *in vivo* Schirmer’s test after exposure to different concentrations of FA, while the rest twenty-one rabbits served as cell sources for further *in vitro* experiment. The study design was privileged by direct match of the *in vivo* and *in vitro* results within the same species. This study was carried out in strict accordance with the recommendations in the Guide for the Care and Use of Laboratory Animals of the National Institutes of Health. The protocol was approved by Institutional Animal Care and Use Committee of the Chang-Gung Memorial Hospital. (Permit Number: 2008110601). All surgery and interventions were performed under ketamine anesthesia, and all efforts were made to minimize suffering. After rabbit was sacrificed by lethal pentobarbital injection, the eyes were proptosed and the corneas were extracted by circumferential excision 2 mm posterior to corneoscleral junction.

### Cornea Epithelium Culture *ex vivo*

After the trephination of the donor cornea, the Descemet’s membrane was removed to prevent contaminating cornea endothelium. The cornea was then cut into 24–36 pieces and carefully placed on the culture dish with epithelial side down in the culture media (DMEM containing 10% FBS and 100 mg/ml penicillin - streptomycin) and incubated at 37°C. After immersed in the culture media, the cornea epithelial cells grew out from the cornea tissue onto the culture disc. The media were changed every other day for good epithelial cell growth until 95% confluence.

### Formaldehyde Solutions

FA solutions (Longtek Scientific Co Ltd, Hsin-Chu, Taiwan) were made from 37% formaldehyde (12.3 M) diluted in sterile distilled water to make 5 ppm (0.16 mM), 10 ppm (0.33 mM), 20 ppm (0.67 mM), 50 ppm (1.6 mM), 100 ppm (3.3 mM), 150 ppm (5 mM), 200 ppm (6.7 mM), 300 ppm (10 mM), 400 ppm (13.3 mM) and 600 ppm (20 mM). The ppm value was provided by the manufacturer. The diluted FA solutions were distributed in aliquot of 10 ml vials and sealed with no air space. All FA solutions were kept at 4°C until used. For treatment, each FA solution was used directly on the cultured cells or tissues only once and discarded.

### Formaldehyde Exposure

Confluent epithelial cells after passage one were exposed to FA. The culture media were replaced with different concentrations of FA solution (0–600 ppm) for short period (3–5 min) or with 100 ppm FA for different exposure time (0–30 min). The cells were washed in order to remove the residual formaldehyde and incubated in culture media without FA until evaluation.

### Cell Morphology Changes Evaluated by Grading System

Cells were exposed to 0, 5, 10, 20, 100, 200, 400 and 600 ppm of FA for 3 minutes, washed with BSS for 3 times to remove formaldehyde, followed by continuous incubation in culture media for 1 day and 7 days without FA. Cell morphology was evaluated according to the listed guideline of the grading system (Table 1). Grade 0 indicates no cell toxicity; round cells are less than 5% (<5%) and cells are well attached onto disc. Grade I indicates very low cell toxicity; round cells are less than 20% (<20%) and loosely attached cells are less than 10% (<10%). Grade II indicates mild cell toxicity; round cells are less than 50% (<50%). Grade III indicates moderate cell toxicity; round cells are less than 70% (<70%) and the level of cell rupture is greater than 30% (>30%). Grade IV indicates severe cell toxicity; round cells are greater than 70% (>70%) and almost all cells are damaged.

**Table 1:** Grading system for cell morphology examined under microscope.

Grade	Cell toxicity	Cellular morphology in high power field (HPF, 10×40)
0	No	Round cells <5%; cells well attached onto disc
I	Very mild	Round cells <20%; loosely attached cells <10%
II	Mild	Round cells <50%
III	Moderate	Round cells <70%; ruptured cells >30%
IV	Severe	Round cells >70%; almost all cells damaged

### Cell Survival Rate Determination

The corneal epithelial cells were treated with 0, 5, 10, 20, 100, 200, 400 and 600 ppm FA for 3 minutes, washed as before to remove residual FA and incubated in culture media for 7 days after FA removal. The cells were then trypsinized, stained with trypan blue and counted under microscope for viability percentage. Dead cells would take up dyes and were stained blue and surviving cells would be refractive without taking up dyes. Every test was repeated three times. The survival rate was calculated as the percentage of surviving cells divided by the total number (dead and living) of cells.

### MTT Assay for Cell Proliferation

Cell survival was also measured at 570 nm using a commercial kit (Vybrant MTT Cell Proliferation Assay Kit, Molecular Probes, Eugene, OR). This is a modified Mosmann’s method to measure the viable cells with a tetrazolium salt 3-(4,5-dimethylthiazol-2-yl)-2,5-diphenyltetrazolium bromide (MTT) reagent [13]. The assay followed the manufacturer’s protocol. Briefly, rabbit corneal epithelial cells were cultured in a 96-well plate and treated with 0, 5, 10, 20, 100, 200, 400 and 600 ppm FA for 3 minutes, excess FA was removed by washing as before, and cells were incubated for 24 hours without FA. Cells were then treated with 20 µl reagent in Dulbeccos’ PBS (pH6.0) for 2 hours at 37°C in a humidified CO_2_ incubator. The absorbance of the solubilized formazan product was determined at 570 nm using an ELISA reader (Multiskan RC, Labsystems, Helsinki, Finland). The control untreated corneal cells were designated as 100%, test samples were evaluated as percentage of the control.

### Cell Death Assay by Flow Cytometry

Fluorescein labeled (FITC) Annexin V/PI Apoptosis kit (Biosciences Pharmingen, San Diego, CA) and PI staining for cell cycle were used for the assay. Briefly, cultured cornea epithelial cells were treated with different concentrations of FA for 3 min and the cells were washed as before to remove residual FA and incubated in culture media. The cells were harvested 4 hours after and resuspended in binding buffer (10 mM Hepes/NaOH, pH 7.4, 140 mM NaCl, 2.5 mM CaCl_2_) containing Annexin V-FITC (1 pg/ml Annexin V) and PI (1 pg/ml) or PI only. The mixture was incubated for 10–15 min at room temperature in dark and analyzed on flow cytometer.

### Mitochondria Changes by Fluorescence Labeling

MitoTracker^R^ kit (Invitrogen, Life Technology, Carlsbad, CA) was used to label mitochondria. The kit contains the dye which passively diffuses across the plasma membrane and accumulates in active mitochondria. The experimental procedure followed manufacturer’s protocol. Briefly, the MitoTracer^R^ was diluted to working concentrations of 100–500 nM. Cells were grown on coverslips inside a Petri dish filled with the culture medium. When cells reached 70% confluence, Cells were treated with 0, 5, 20 and 600 ppm FA for 10 min. The medium was removed from the dish and prewarmed (37°C) staining solution containing MitoTracker^R^ probe was added for 4 hours. After staining, cells were washed with fresh, pre-warmed buffer and observed under the fluorescence microscope.

### *In vivo* Schirmer’s Test

This test was performed on New Zealand rabbits and used a commercial kit of Eagle Vision Color Bar Schirmer’s tear test (Eagle Vision, Inc., Memphis, TN). Briefly, small round filter paper disc (1 cm in diameter) soaked in 0, 20, 100, 200 or 300 ppm FA was applied to the rabbit eye and the eye was closed for 5 minutes. The eye was subsequently washed with BSS to remove residual FA. Gentamycin ointment was applied to the eye two times daily for the experimental period. At 1, 3, 7 and 10 days after FA exposure, the filter strip was then inserted to the rabbit eye and the eye was closed for 5 minutes. Moisture on the charted filter strip appeared as blue color. The amount of moisture was measured by how far the blue dye traveled on Schirmer’s paper. Both eyes were tested at the same time with one eye as control and the other as test.

### Western Blotting

We evaluated the effect of ERK and JNK activation on FA exposure to the corneal and conjunctiva tissue in New Zealand rabbits. The rabbit eyes were exposed to round filter paper disc (1 cm in diameter) with 0, 50, 100, 150 or 200 ppm FA for 5 minutes. The eyes were washed with BSS and treated with gentamycin ointment as before until evaluated. The cells of corneal epithelium and conjunctiva were explanted two months after exposure. The explanted corneal epithelial cells and conjunctiva cells were trypsinized, lysed and examined by western blotting. Western blotting was performed using 25–50 µg of protein per lane. The polyethersulfone polyvinylidene fluoride membranes containing the proteins were incubated with rabbit antibodies against JNK/pJNK and ERK2/pERK2 (Santa Cruz Biotechnology, Santa Cruz, CA) for 2 hours at room temperature. Detection used horseradish peroxidase - conjugated goat anti-rabbit immunoglobulin G (Cell Signaling, Beverly, MA) and an enhanced chemiluminescence reagent (ECL, Amersham, Piscataway, NJ), followed by digital image acquisition and analysis (Image Station Model 2000R; Eastman Kodak, New Haven, CT).

### Statistical Analysis

All tests were performed at least three separate times. Control values were designated as 100%. Statistical significances were determined by Mann - Whitney U test using SPSS ver 13.0 (SPSS Inc, Chicago, IL) for cell survival rates and Sigma Plot 10 for cell count and Schirmer’s tests. Statistics for western blots and flow cytometry used student *t* test. Differences with a *P* value of less than 0.05 were considered statistically significant [14].

## Results

### High Formaldehyde Concentration Induced Abnormal Cell Morphology Changes

**Figure 1:**
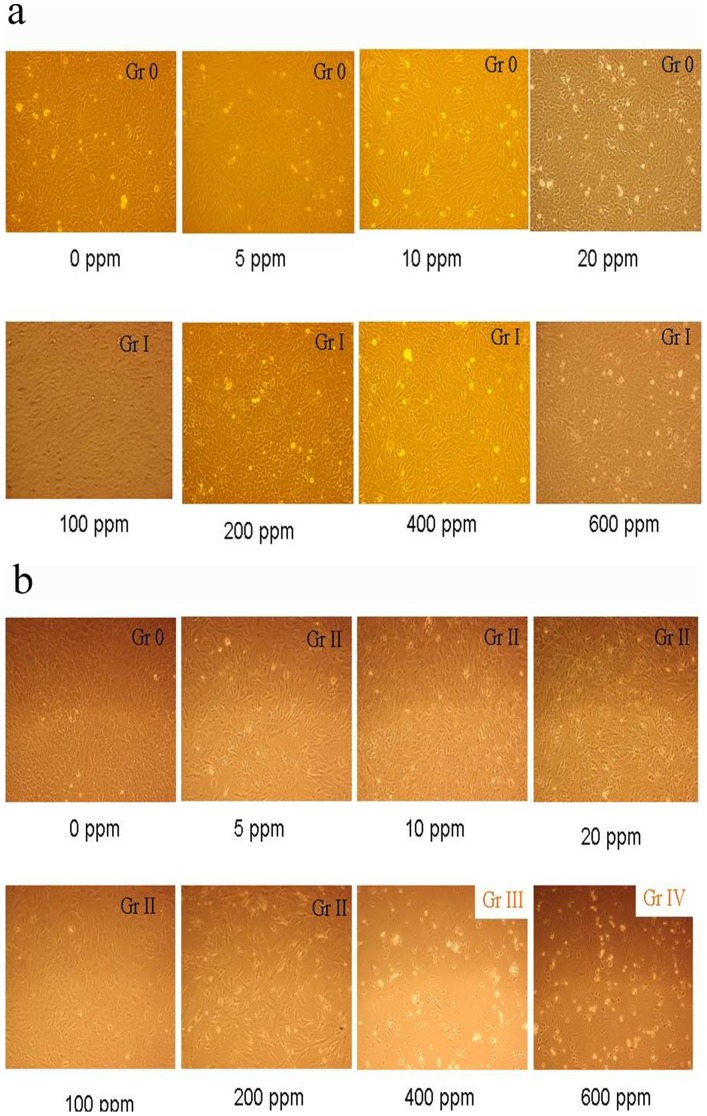
Cell morphology in rabbit corneal epithelial cells exposed to various FA concentrations for 3 minutes and examined at one day (a) and seven day (b) after exposure. Grades (Gr) indicated are according to the grading system listed in Table 1.

The grading system is listed in Table 1. One day after 3 minutes (min) FA exposure, the cell morphology in groups treated with 5, 10 and 20 ppm FA showed grade 0; while the groups treated with 100, 200, 400 and 600 ppm exhibited grade I damage (Fig. 1a). Seven days after the 3 min exposure, the control group was found with normal nuclear and cytoplasm ratio (N/C ratio, grade 0); the exposed groups showed sub-confluent growth with normal N/C ratio, and less than 10 cells were dead (grade II) for 5, 10, 100 and 200 ppm FA treated groups under the high power microscope examination. Some of the cornea epithelial cells were found with fibroblast transformation and the cell membrane showed spindle shape. At 400 ppm FA exposure, the cells became round and decreased N/C ratio (grade III), and detached from the culture disc plate. At 600 ppm FA exposure, cells were detached and showed abnormal N/C ratio (grade IV) (Fig. 1b). These were summarized in Table 2. The results indicated that short time (3 min) exposure to FA did not show visible cellular morphology changes for low FA concentrations (up to 20 ppm) at 1-day observation; however, longer period (7-day) observation did show grade II morphological changes even for 5 ppm FA exposure.

**Table 2:** Grading for cells treated with FMD.

Formaldehyde (ppm)	0	5	10	20	100	200	400	600
Day 1	0	0	0	0	I	I	I	I
Day 7	0	II	II	II	II	II	III	IV

### Cell Viability Assay Showed that the Formaldehyde Contact Reduced Cell Survival Rate

**Figure 2:**
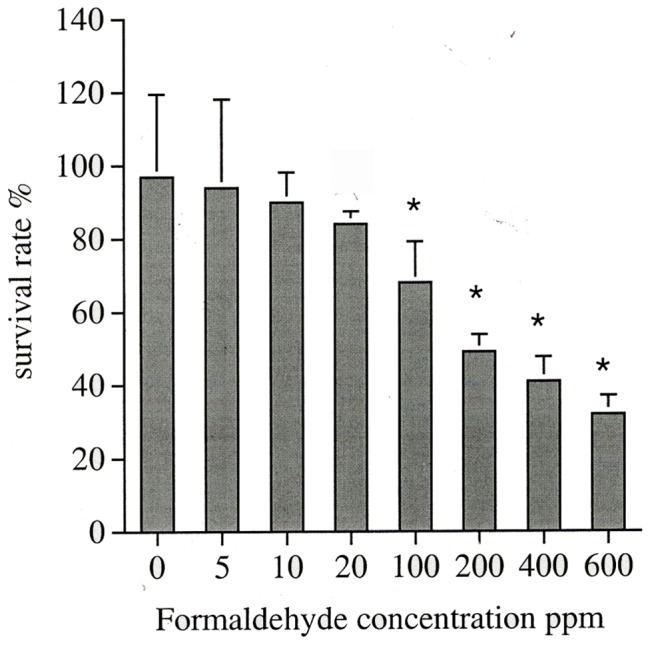
Cell survival rate (%) of rabbit corneal epithelial cells exposed to various FA concentrations for 3 minutes and examined at 7 days after exposure. Percentage indicates the ratio of surviving cells to total cells in the same dish. Vertical bars indicate standard deviation of the mean. [*] represents P<0.05 as compared to the control group.

The cell survival rate of rabbit corneal epithelium seven days after 3-min exposure to different concentrations of FA showed a concentration-dependent cell death. The cell survival rates were 97±22.47%, 94±24.2%, 90±8.2%, 84±45%, 68±11.16%, 49±4.63%, 41±6.61% and 32±5.09% (mean ± standard deviation of the mean) for the group exposed to 0, 5, 10, 20, 100, 200, 400 and 600 ppm FA, respectively. When rabbit corneal epithelial cells were treated with greater than 200 ppm FA, the cell survival rates were all less than 50% (Fig. 2). Although the treated cells of up to 100 ppm FA seemed to tolerate well for a short time and did not show statistically significant difference from the control, a trend of decreased survival rate was apparent at 7-day observation. This indicated that even changing the culture media frequently and feeding with fresh nutrient did not renew or enhance the growth of the epithelium. The damage from FA toxicity seemed long lasting.

**Figure 3:**
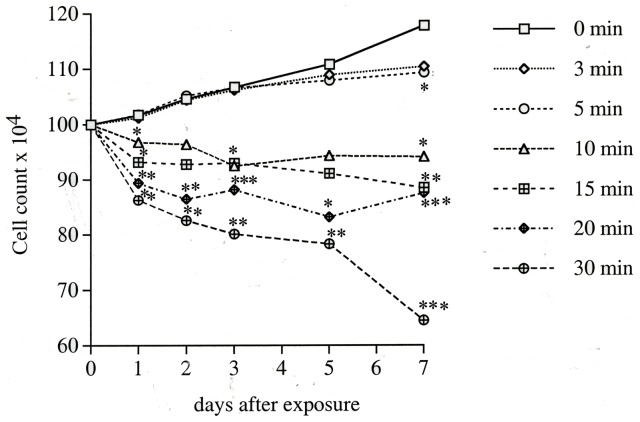
Cell numbers of rabbit corneal epithelial cells exposed to 100 ppm for various times (0–30 min) and examined with trypan blue staining at indicated time intervals (1–7 day) after exposure. Statistical comparisons with control are indicated as [*] for P<0.05, [**] for P<0.01 and [***] for P<0.001.

To examine the cell number changes in relation to exposure time and observation time after exposure, the rabbit cornea epithelial cells were exposed to 100 ppm FA for 3, 5, 10, 15, 20 and 30 min and the cell numbers were examined daily for 7 days after FA exposure. The cell number increased to 118%, 110% and 109% for control, 3 min-, and 5 min- treatment group, respectively, while longer time exposure decreased the cell number to 94%, 89%, 88% and 65% for the 10 min, 15 min, 20 min and 30 min treatment groups, respectively (Fig. 3). Although the 3 min and 5 min exposure showed increased cell number, however, the increase was not as much as control at 5 to 7-day post exposure. Statistically significant differences (P<0.05) were observed for 10 min and 15 min treatment groups at days 1, 3, 7 and for the 20 min and 30 min treatment groups at days 1, 2, 3, 5, 7 as compared to the control group. The results indicated that lower exposure time (3–5 min) did show increased cell number as in the control group. However the cell proliferation rate dropped after 5 days post treatment, although the cell numbers were exceeded the starting cell number.

**Figure 4:**
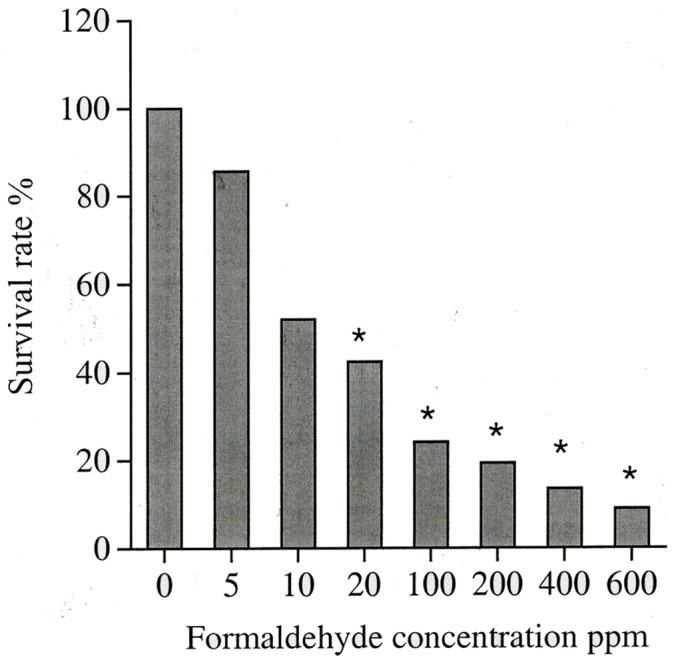
MTT assay for cell viability by colorimetric method. The rabbit cornea epithelial cells were exposed to various concentrations of FA for 3 minutes and the cell survival rates were evaluated 24 hours after exposure. [*] denotes P<0.05 as compared to the control group.

The MTT assay, which measures the cell proliferation activity, revealed that the viable cells decreased significantly after three-minute transient FA exposure at concentration of greater than 10 ppm and the damage seemed long lasting even after seven days post treatment. The cell survival rates were 100±0.17%, 85.71±0.06%, 51.98±0.15%, 42.46±0.11%, 24.21±0.04%, 19.44±0.06%, 13.49±0.05%, and 9.01±0.02% for the groups of control, 5, 10, 20, 100, 200, 400 and 600 ppm FA treated group, respectively. The statistically significant differences were observed between the control group and the groups of FA treatment with concentration above 20 ppm (p<0.05). (Fig. 4). For the lower FA dose (5–10 ppm), the difference from the control was not statistically significant; however, the trend of decreased survival rate was apparent.

### Corneal Epithelial Cells Displayed Apoptosis, Necrosis and Mitochondria Clustering around the Cell Nucleus after Transient Formaldehyde Treatment

**Figure 5:**
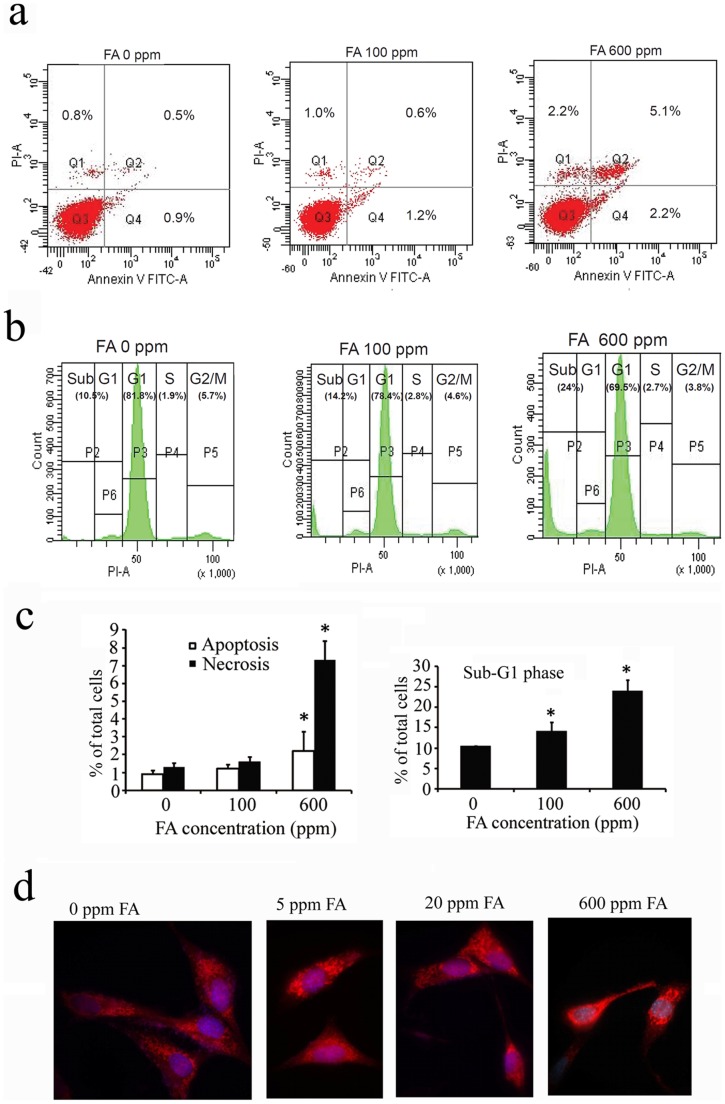
Representative graphs of flow cytometry assays are shown in (a) and (b). In the FITC-Annexin V/PI labeling experiment (a), the corneal epithelial cells were treated with 0, 100, 600 ppm FA for 3 min and examined 4 hours later. Apoptotic (G4 region) and necrotic (G1 and G2 regions) cells appear increased in FA treated cells as compared with the control (0 ppm). In the cell cycle examination (b), FA-treated cells increase at subG1 phase as compared to the control (0 ppm). Bar graphs (c) depict the statistical results of 3 assays. Vertical bar indicates standard deviation of the mean. The [*] indicates P<0.05 as compared to the control (0 ppm) cells. d) Mitotracker staining assay revealed that the mitochondria (bright red) accumulate near the perinuclear region after FA treatment.

The flow cytometry results revealed that apoptosis and necrosis (both secondary and primary necrosis) were increased in the treated cells (Fig. 5a). Accumulation of subG1 phase in the cell cycle analysis was evident for the cells treated with 100 ppm and 600 ppm FA (Fig. 5b). Statistical analysis revealed that significant increase in apoptotic/necrotic cells and accumulation of sub-G1 phase were apparent in the FA treated cells (Fig. 5c). The FA treatment also caused mitochondria clustering around the cell nucleus (Fig. 5d).

### Prolonged Tear Production Increase after Transient Eye Exposure to Formaldehyde Measured by Schirmer’s Test

**Figure 6:**
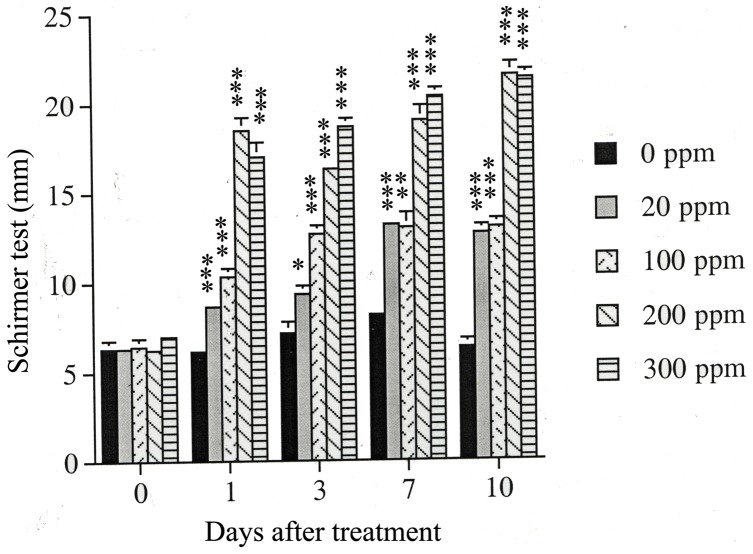
Schirmer’s test measured in New Zealand rabbits (in vivo) after exposing the rabbit eye to various FA concentrations (0–600 ppm) for 5 minutes and examined at indicated time after exposure. Vertical bars denote standard deviation of the mean. [*] indicates P<0.05, [**] for P<0.01 and [***] for P<0.001 as compared to the respective control group.

To evaluate whether FA treatment affected basic tear function *in vivo*, Schirmer’s test was used on the eyes of New Zealand rabbits after FA exposure at concentrations of 20, 100, 200 and 300 ppm for 5 min and examined at days 0, 1, 3, 7, and 10 after exposure. No change was observed for control animal while increased tear production was observed for FA-treated animals irrelevant to what FA concentration used and what days-after observed (Fig. 6).

### Down-regulation of ERK and Persistent JNK Activation in Corneal and Conjunctiva Cells on Formaldehyde Exposure in Animal Model

**Figure 7:**
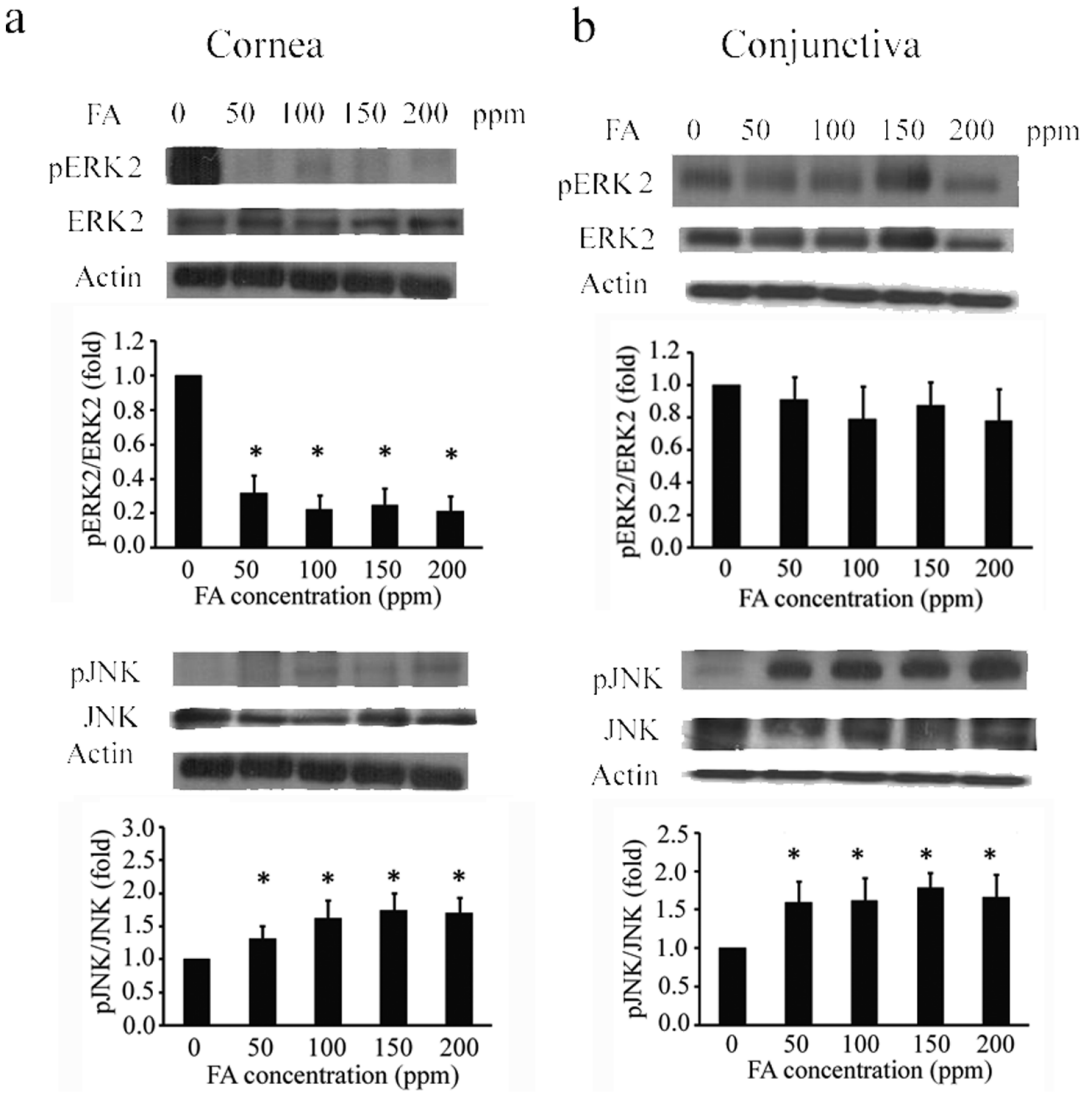
Western blots and the bar graphs of ratios for ERK2/pERK2 and JNK/pJNK in corneal epithelial cells (a) and conjunctiva explanted from rabbit eyes (b) treated with various concentrations (0–200 ppm) of FA for 5 minutes ***in vivo*** and examined at 2 months after treatment. The pERK2 and pJNK indicates phosphorylated ERK2 and phosphorylated JNK, respectively. ERK2 and JNK denote total ERK2 and total JNK, respectively. Actin is used as an internal control. Vertical bar indicates standard deviation of the mean. [*] indicates P<0.05 as compared to the control (0 ppm) group.

The phosphorylation of ERK and JNK in corneal and conjunctiva tissues after 50, 200, 150 and 200 ppm FA exposure was evaluated by western blotting 2 months after exposure. For the corneal cells, the treatment resulted in markedly decreased ERK phosphorylation and increased JNK phosphorylation (Fig. 7a), while in the conjunctiva cells, the decrease in ERK phosphorylation was slight, but the increase in JNK phosphorylation was obvious in the treated group (Fig. 7b). The results indicated that transient exposure to FA caused down-regulation of ERK activation and up-regulation of JNK activation in corneal tissue, as well as persistent JNK activation in conjunctiva for the long-term (2 months) observation.

## Discussion

FA is classified as carcinogen and is ubiquitously present in our living environment. In the anatomy laboratory, FA is used to preserve human body as teaching material. FA has been reported as cytotoxic and apoptotic on PC12 cells [9]. Exposure to FA, especially in male subjects, can cause reproductive toxicity [6]. As FA is highly volatile, most studies are related to respiratory exposure. Effect to the eye tissue has been studied using direct contact with 37% FA which caused drastic injury [11], [12]. Evaluation of different FA concentrations and different exposure time to the cornea has not been reported in the literature. In order to establish an animal model for eye research, we used rabbit eye and the chemical formaldehyde to examine eye injury.

We have evaluated the FA toxic effect on the cornea and the conjunctiva for different concentrations and different exposure duration. The cell morphology observed at day 1 after 3 min exposure did not show changes up to 100 ppm and at higher FA concentrations up to 600 ppm only showed very mild changes (grade II). However, at day 7, even exposure to 5 ppm, cells showed very mild changes (grade II) up to 200 ppm and greater damages were observed above 400 ppm exposure. The cell count also showed significant decrease in cell number for 3 min above 100 ppm exposure at day 7 (Fig. 2). We therefore used 100 ppm FA to check the time exposure experiment. The exposure time up to 5 min and observed for 7 days showed limited cell proliferation as shown in Fig. 3. Exposure for 10 min or longer did show significant decrease in proliferation. MTT assay detects living but not dead cells and the end product generated is dependent on the degree of activation by the living cells. The method measures cytotoxicity and proliferation of the cells and is evaluated by spetrophotometry. It is more sensitive than the trypan blue assay and we detected a significant decrease in cell survival with more than 20 ppm FA treatment. Mitochondria distribution in the cell may reflect the energy requirement for that cellular compartment. The accumulation of mitochondria at perinuclear region after FA treatment probably associates with cell survival under stress condition. The FITC-Annexin V/PI labeling indicated that cells transiently exposed to 100 ppm FA (3 min) increased percentage of apoptotic and necrotic cells as shown in Fig. 5a. Higher concentration of FA (600 ppm) induced more severe cell death. The treated cells also showed accumulation at sub-G1 phase of cell cycle indicating the cell death involved G1 phase inhibition and cell membrane damage. Our long term observation demonstrated that irreversible cell damage from transient FA exposure was obvious.

Our results also showed that transient treatment with FA for 3 min, the corneal epithelial cells were transformed to fibroblastic morphology at seven-day culture. Activated fibroblasts are key contributors to the accumulation of extracellular fibrous matrix [15]. Once fibrosis occurred, tissue could produce excessive interstitial collagen and extracellular matrix [16] indicating that the corneal epithelium may proceed to tissue fibrosis. There is a potential for epithelial cells differentiating into fibroblastic cells in response to morphogenic pressure from injured tissue. The phenomenon is known as epithelial – mesenchymal transition and it can occur in limbal location in rabbit corneal explant [17].

Abnormal sub-epithelial fibrosis and epithelial keratinization, such as severe ocular surface fibrosis, can cause vision impairment [18], [19]. Several inflammatory cytokines may initiate the mechanism of corneal subepithelial fibrosis through corneal stromal fibroblast activation [20], [21]. FA was reported to significantly increase IL-8 and IL-1β production in cells stimulated with phorbol 12- myristate 13-acetate [22]. Transient FA exposure may increase the possibility of corneal fibrosis.

Many evidences have demonstrated that the MAPK family of serine/threonine kinases in the regulation of cell proliferation, differentiation, and apoptosis. In response to a stimulus, MAPK kinase kinase can phosphorylate a specific MAPK kinase, that in turn phosphorylates its specific downstream substrates. ERKs (p44/42 MAPK or ERK1/2) are phosphorylated by the sequential activation of RAF1 and MEK1/2 in response to growth factors and mitogens and induce either proliferation or differentiation [23], [24]. Phosphorylation of JNK (or SAPK) occurs in response to the selective activation of a MAP kinase kinase such as MEKK1 [25], ASK1 [26] or MLKs [27] followed by phosphorylation of either MKK4 [25] or MKK7 [28]. This pathway is stimulated by environmental and chemical stress as well as by exposure to cytokines, and it appears to play a role inducing the apoptosis [29], [30]. Studies have demonstrated that ERK activation controls cell survival.

This study focused on the corneal cells which are vulnerable for damage, while the conjunctiva cells served as a control to illustrate different response from corneal cells. As in *in vivo* experiment, FA disc were applied to the cornea, the conjunctiva cells were also affected due to contact with the FA. Unlike the conjunctiva tissue which has blood supply, the corneal tissue receives no direct blood flow. The response to injury in conjunctiva for signaling molecules would be more obvious than the cornea. In this report, we also included the conjunctiva for signaling molecule studies. Our results demonstrated that long term (2 months) after transient FA exposure, JNK was persistently activated in conjunctiva, while in the corneal tissue, the ERK inhibition and JNK activation were apparent. The slight ERK changes in conjunctiva may reflect possible tissue repair due to the blood flow. Wang and Lu [31] have demonstrated that JNK activation is one of the mechanisms to control apoptosis in corneal epithelial cells. UV-irradiation-induced corneal epithelial cell apoptosis is mediated through activation of the SAPK/JNK signaling pathway [32], [33]. Our results suggested that the ERK inhibition and JNK activation control the fate of corneal epithelium and persistent JNK activation caused the cornea and conjunctiva tissue damage long after the transient FA exposure. This could program the corneal and the conjunctiva tissues to enter apoptotic and necrotic pathway which we have observed with flowcytometry. In addition, FA treatment also caused the cell cycle changes with accumulation at sub-G1 phase.

### Conclusions

The results indicated that transiently direct contact with FA even as low as 5 ppm induced damage in corneal epithelium cells *in vitro* and *ex vivo.* Lower FA concentration exposure may not show clear damage at short-time evaluation, but longer period after exposure, the cornea epithelial cells showed abnormal cell count, cell morphology and tear production. Apoptosis/necrosis and cell cycle retardation at G1 phase were apparent. As in close contact with cornea, conjunctiva cells were also affected.
